# Shared barriers and facilitators to enrollment of adolescents and young adults on cancer clinical trials

**DOI:** 10.1038/s41598-022-07703-5

**Published:** 2022-03-09

**Authors:** Nupur Mittal, Aniket Saha, Viswatej Avutu, Varun Monga, David R. Freyer, Michael Roth

**Affiliations:** 1 Department of Pediatrics, Rush University Medical Center, Chicago, IL USA; 2 Department of Pediatrics, Prisma Health Children’s Hospital, Greenville, SC USA; 3 Department of Medicine, Memorial Sloan Kettering Cancer Center, New York, NY USA; 4 Division of Medical Oncology, Department of Internal Medicine, University of Iowa, Iowa City, IA USA; 5 Department of Pediatrics, Keck School of Medicine, University of Southern California, Los Angeles, CA USA; 6 Division of Pediatrics, The University of Texas MD Anderson Cancer Center, 1515 Holcombe Blvd., Houston, TX 77030 USA

**Keywords:** Health care, Oncology

## Abstract

Adolescent and young adult (AYA) enrollment in cancer clinical trials (CCT) is suboptimal. Few studies have explored site level barriers and facilitators to AYA enrollment on CCTs and the efficacy of interventions to enhance enrollment. A cross sectional survey was developed by the COG AYA Oncology Discipline Committee Responsible Investigator (RI) Network to identify perceived barriers and facilitators to enrollment, as well as opportunities to improve enrollment. Associations of barriers and facilitators to enrollment with program demographics were assessed. The survey was sent to all AYA RI Network members (n = 143) and quantitative and thematic analyses were conducted. The overall response rate was 42% (n = 60/143). Participants represented diverse institutions based on size, presence or absence of dedicated AYA programs, and proximity and relationship between pediatric and medical oncology practices within the institution. The most frequently cited barriers to enrolling AYAs in CCTs were administrative logistical issues (45%), disparate enrollment practices (42%) and communication issues (27%) between pediatric and medical oncology and perceived limited trial availability (27%). The most frequently reported facilitators to enrollment included having strong communication between pediatric and medical oncology (48%), having a supportive research infrastructure (35%) and the presence of AYA champions (33%). Many barriers and facilitators were similar across institutions and AYA program types. Shared barriers and facilitators to AYA CCT enrollment exist across the landscape of cancer care settings. Interventions aimed at increasing coordination between pediatric and medical oncology clinical trials offices and providers have high potential to improve site-level AYA enrollment.

## Introduction

Cancer clinical trials (CCTs) are vital for studying disease biology and improving survival and health-related quality-of-life outcomes; however, only 2–5% of all AYAs with cancer enroll in a CCT^1,2^. Despite a growing focus on addressing disparities in AYA cancer care and outcomes, few studies have assessed factors contributing to the low enrollment of AYAs into CCTs. Even fewer studies have assessed the efficacy of interventions to improve enrollment. AYA cancer biology, tolerance to intensive treatment and survival outcomes for specific malignancies differ in comparison with older adults and younger children, strongly supporting the need to identify optimal treatment and supportive care approaches in the AYA population^3,4^.

The reasons for limited AYA CCT enrollment, even amongst the ones eligible for trials while not well understood, have been hypothesized in recent reviews^5–7^. These include global issues such as the perception of limited availability of relevant CCTs, regional issues such as lack of referral of AYA patients to centers with National Cancer Institute (NCI)-CCTs and institutional-level issues such as not activating CCTs due to the regulatory burden and cost of study activation and conduct. Additional suggested barriers at the site level include lack of eligibility screening procedures, limited communication between medical and pediatric oncologists, limited knowledge and comfort with other NCI Clinical Trials Network (NCTN)-CCTs, as well as time and economic constraints to open and enroll AYAs on CCTs. In addition psychosocial barriers such as stress/distress, developmental and emotional maturity, feeling ill, experimentation play a role as well^8–12^. While many publications have proposed potential barriers to enrollment, data reporting actual barriers are sparse^8–12^.

In 2018, the Children’s Oncology Group (COG) AYA Oncology Discipline Committee developed an international network of AYA Responsible Investigators (RIs) consisting of > 140 individuals from demographically and geographically diverse sites that serve a variety of distinct roles at their respective institutions such as physicians, nurse practitioners, nurse navigators and research staff. Institutions included free-standing children's hospitals, sites with pediatric and medical oncology on shared or separate campuses, and sites located in community and urban settings.

The primary purpose of the AYA RIs is to optimize AYA enrollment onto COG-led trials, and other NCTN trials in which COG is participating, at their sites. AYA RI responsibilities are focused on implementing steps to facilitate clinical trial enrollment of AYAs treated within their institution as previously described^13^. The primary mechanism through which the AYA RI Network supports enrollment is providing education and peer support to institutional AYA RIs. To achieve its goal, the AYA RI Network hosted a series of informal webinars allowing RIs to share barriers and facilitators to AYA enrollment, as well as unique challenges to AYA enrollment based on site-specific factors. The purpose of this survey was to systematically identify shared barriers and facilitators to AYA accrual in CCTs as reported by members of the COG AYA RI Network and assess associations with program demographics to inform institutional and national interventions aimed at increasing AYA CCT enrollment.

## Methods

### Survey design, development, and setting

A cross sectional survey was developed by the COG AYA Oncology Discipline Committee RI Network leadership (AS, NM, MR, DF) to identify perceived barriers and facilitators to enrollment, as well as opportunities to improve enrollment. The survey was piloted with five AYA oncology stakeholders and revised as needed for content, readability and clarity. The survey consisted of two parts: the first comprised twelve questions on demographic information, including program characteristics, institutional structure and the relationship between pediatric and medical oncologists; the second comprised four free text questions on participants’ perspective on: (1) institutional barriers to enrollment; (2) facilitators to enrollment; and (3) possible solutions to improve AYA accrual to CCTs at the institutional level; and (4) cooperative group level. Of note, participants could have their responses placed into multiple categories and multiple answers were permitted (Supplemental Table 1).

### Survey distribution

The online survey was administered and responses were stored using REDCap. An email was sent to all COG AYA RI Network members (n = 143) with a brief description of the study and an embedded, clickable link to the survey. One RI from each institution in the RI Network was sent the survey. In case of non-response, the survey was redistributed two additional times within 4 weeks from initial survey distribution (December 2019–January 2020). All methods were carried out in accordance with relevant guidelines and regulations. The study was approved as exempt by the Prisma Health Upstate IRB. Participation in the survey was voluntary and signed informed consent was not obtained for individual participants as it would be the only link between the participant responses and their identity.

### Data analysis

Demographic information was summarized for all respondents. Response frequencies and proportion of total responses were calculated for all categorical variables. Means and medians were calculated for all continuous variables. Responses to the free text questions were reviewed and themes for each of the four free text questions were identified by two study investigators (NM, AS). The themes were reviewed and consensus was achieved among three study investigators (NM, AS, MR). For each question, responses were subsequently independently categorized into the previously agreed upon themes (NM, AS). When there was disagreement in the categorization of responses, agreement was sought between the two raters (NM, AS); if consensus was not reached, a third, independent investigator (MR) categorized the response. Fisher exact test and Chi Square analysis (GraphPad Prism, San Diego, CA) were used to assess the association between demographic variables and perceived barriers and facilitators to enrollment. Two-sided p value < 0.05 was considered statistically significant.

## Results

### Respondent and institutional demographics

The overall survey response rate was 42% (n = 60/143) and 97% of these respondents (n = 58) completed the entire survey. The participants represented a diverse group of institutions based on size, presence of an AYA Program and geographic proximity between pediatric and medical oncology (Table 1). Approximately one-third (n = 22) of respondents reported that their institution saw > 100 new AYA patients annually. The percent of respondents that categorized their institution as a children’s hospital within an adult medical center, free standing children’s hospital, and community hospital was 50% (n = 29), 36% (n = 21) and 12% (n = 7), respectively.

**Table 1:** Institutional and AYA program characteristics.

Question	Response	N (% of responders)
AYA program existence	Yes/in development	45 (75%)
	No	15 (25%)
New AYA patients/yr at institution	< 100	38 (63%)
	> 100	22 (37%)
AYA program size (new patients/yr in past 3 yrs)	< 100	17 (57%)
	> 100	13 (43%)
AYA services provided	Cancer treatment	23 (77%)
	Genetic counseling	18 (60%)
	Oncofertility	29 (97%)
	Psychosocial support	28 (93%)
	Sexual health	15 (50%)
	Survivorship care	21 (70%)
	Symptom management	20 (67%)
Cancer type served	All cancers	26 (87%)
	Limited	4 (13%)
Institution type	Free standing CH	21 (36%)
	CH within adult medical center	29 (50%)
	Community hospital	7 (12%)
	Academic medical center	18 (31%)
Geographic proximity (Ped/Med oncology)	Same building	14 (24%)
	Same campus	28 (48%)
	Different campus	11 (19%)
Single institutional IRB	Yes	43 (74%)
	No	12 (21%)
Joint tumor boards	Yes (all/some diagnosis)	25 (43%)
	No	33 (57%)
Cross-department ad hoc AYA Discussions	Yes	52 (90%)
	No	6 (10%)
Med oncology enroll onto COG trials	Frequently/occasionally	26 (45%)
	Rarely/never	24 (41%)
	No Med oncology at institution	8 (14%)

### AYA program demographics

Of the 60 responders, three quarters reported having an active AYA program (n = 31) or one in development (n = 14) (Table 1). Approximately 43% of those with active AYA programs (n = 13) care for > 100 AYAs each year. The majority of active AYA programs (n = 26, 87%) cared for patients across all cancer diagnoses as opposed to being focused on a specific diagnosis. Services offered within active AYA programs varied with almost all providing onco-fertility services (n = 29, 97%) and psychosocial support (n = 28, 93%).

### Relationship between pediatric and medical oncology

With regards to geographic proximity, 24% of respondents (n = 14) reported that pediatric and medical oncology providers work in the same building, 48% (n = 28) were located on the same campus and 19% (n = 11) were located on separate campuses. Three quarters of respondents (n = 43) stated they had a single IRB at their institution to approve both pediatric and medical oncology trials. Forty-three percent (n = 25) of respondents stated they had formal joint tumor boards between pediatric and medical oncology for all or some oncologic diagnoses. Almost all participants (n = 52, 90%) reported having ad hoc discussions with their medical oncology colleagues about AYA patients. When asked how often their medical oncology colleagues enrolled their patients onto COG trials, 45% (n = 26) responded that this occurred ‘frequently/occasionally,’ while 41% (n = 24) responded that this ‘rarely/never’ occurred.

### Barriers to enrollment

**Figure 1:**
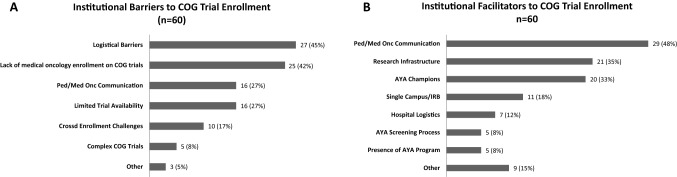
Reported institutional barriers (**A**) and facilitators (**B**) to enrollment of AYA patients onto COG trials.

Participants identified several perceived barriers to enrolling AYAs at their local sites with 102 total responses provided by 60 participants (Fig. 1A). The level of agreement on categorization on first review and reconciliation was 86% and 98%, respectively. The remaining 2% of the responses were reconciled by a third reviewer to reach 100% consensus. The most frequently cited barriers to enrolling AYAs included: administrative logistical barriers (45%), perceived lack of interest by medical oncology (42%), communication issues between pediatric and medical oncology (27%) and perceived limited CCT availability (27%). Examples of specific responses categorized as site level barriers are presented in Table 2.

**Table 2:** Reported barriers and facilitators to enrollment.

Question	Response category	Definition	Example of response
1. What are the main barriers to accrual of AYA patients onto COG clinical trials at your institution? If applicable, please include barriers to collaboration with medical oncology for clinical trial accrual in your response	Administrative logistical barriers	Administrative barriers at site level that negatively impact AYA trial enrollment	‘Perceived age barrier by hospital executives’
	Perceived medical oncology lack of interest	Perceived lack of enthusiasm to enroll AYA patients to trials; refusal to transfer care of AYA patients to pediatric oncology	‘Some medical oncologists rather keep patients than refer them if they cannot enroll on trial themselves’
	Cross enrollment challenges	Site level regulatory and structural barriers that hinder AYA patients to be enrolled across cooperative group trials and between medical and pediatric oncology	‘Adult facility is on different campus’
	Pediatric and medical oncology communication issues	Reported negative relationship between medical and pediatric oncology which does not involve regular communication and negatively impacting site level trial enrollment for AYAs	‘Lack of an established pathway for knowledge sharing between pediatric and medical oncologists’
	Limited trial availability	AYA focused clinical trials are limited in availability at site level	‘Adult sites and physicians in Australia not able to participate in COG trials’
	Complexity of COG trials	COG trials are deemed to be too burdensome and complicated at institution by members	‘Perceived complication of COG trials from the medical oncologist point of view – they are often felt to be too complicated and require too many resources to administer in the medical oncology setting’
2. What are the main facilitators to accrual of AYA patients (ages 15–39) onto COG clinical trials at your institution? If applicable, please include facilitators to collaboration with medical oncology for clinical trial accrual in your response	AYA champions	Existence of an individual at institution with focus on AYA clinical trial enrollment at site level	‘We have a champion within the medical oncology group who is able to enroll patients on COG trials’
	Supportive research infrastructure	Presence of a research infrastructure deemed conducive to AYA clinical trial enrollment at site level	‘Strong clinical research infrastructure at my institution allows us to have most non-phase 1 studies open.’
	Good pediatric and medical oncology communication	Reported positive relationship between medical and pediatric oncology involving regular communication positively impacting AYA clinical trial enrollment	‘Dialogue between adult and peds to triage specially to ensure they have availability to open COG clinical trials has been a facilitator’
	AYA screening process	Processes in place at site level that allows patients to be identified as AYAs and screened for available clinical trials at institution	‘Our pediatric Clinical Research Group (GRG) CRAs now screen new patient notifications for potential clinical trial eligibility and maintain a database of patients who are screened’
	Hospital logistics	Administrative policies supporting and allowing AYA Clinical trial enrollment	‘We allow patients to be treated up to age 39 at our Children’s Hospital’
	Presence of formal AYA program	Existence of a dedicated team of individuals at institution providing care to AYA patients	‘AYA program in place with a co-directorship-pediatric oncologist and a medical oncologist.’
	Single campus/IRB	Institutional structured such that there is one campus and single IRB between medical and pediatric oncology	‘Singular IRB and CTSR program allowing more providers to be co investigators on trials’

### Facilitators to enrollment

Participants identified several perceived facilitators to enrolling AYAs in CCTs with 107 total responses provided by 60 respondents (Fig. 1B). The level of agreement on categorization on first review and reconciliation was 77% and 96%, respectively. The remaining 4% were reconciled by a third reviewer to reach 100% consensus. The most frequently reported facilitators to enrollment included strong communication between pediatric and medical oncology (48%), a supportive research infrastructure (35%) and the presence of AYA champions (33%). Specific responses categorized as site level facilitators are presented in Table 2.

### Demographic factors associated with perceived barriers and facilitators to enrollment

We next assessed whether institutional demographics (institution type, presence of a formal AYA program and involvement of medical oncology in COG trial enrollment) were associated with specific perceived barriers and facilitators to enrollment (Supplemental Fig. 1). A strong research infrastructure was more likely to be reported as a facilitator by those institutions if medical and pediatric oncology colleagues were physically in the same building or campus than if they were on a different campus (46.4% vs 0%, p = 0.03). No other variables were significantly associated with differences in perceived barriers and facilitators to enrollment. Similar barriers and facilitators were mostly shared across different types of institutions.

### Desired institutional changes and support from the COG AYA RI network

**Figure 2:**
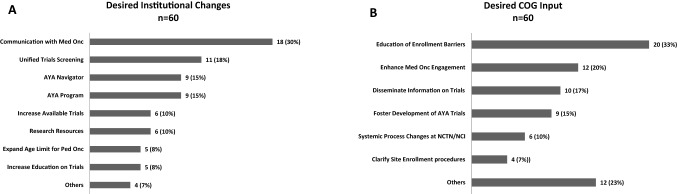
Reported desired changes at the institutional level (**A**) and network group level (**B**) to facilitate and foster AYA clinical trial enrollment.

Respondents noted several changes that could potentially improve local enrollment as shown in Fig. 2A. A total of 73 responses from 60 respondents were reviewed and categorized. The level of agreement on categorization on first review and reconciliation was 77% and 95%, respectively. The remaining 5% were reconciled by a third reviewer to reach 100% consensus. The most frequently reported desired changes included improving communication between pediatric and medical oncology (30%), having a unified approach to CCT screening (18%), having an AYA patient navigator (15%) and having an AYA program (15%). Specific examples of these categories are presented in Table 3.

**Table 3:** Recommendations to improve local AYA enrollment.

Question	Response category	Definition	Example of response
3. If you could change one thing at your institution to increase accrual of AYA patients to COG clinical trials, what would it be?	Unified trial screening	Processes in place at site level that allows patients to be identified as AYAs and screened for available clinical trials at institution with consensus from medical and pediatric oncology	‘We will develop a AYA specific program to oversee the screening process and manage patients once identified’
	AYA navigator	Existence of an individual at institution with focus on helping AYA patients navigate the clinical trial and treatment experience at site level	‘We are working to hire a navigator’
	AYA program	Existence of a dedicated team of individuals at institution providing care to AYA patients	‘Start an AYA clinic with both medical and pediatric oncology’
	Expansion of age limit for pediatric oncology	Institutional policies that allow for non-pediatric patients to be treated by pediatric oncology	‘Convince my administrators that there is not much difference in treating a 21 vs a 25-year-old’
	Improved communication with medical oncology	Reported positive relationship between medical and pediatric oncology involving regular communication positively impacting AYA clinical trial enrollment	‘I would really like to simply increase the interaction between Pediatric and medical oncologist’
	Increase in trial availability	Expansion of number of AYA focused trials at site level	‘More trials available to meet the needs of AYA’
	Research resources	Robust clinical research office and regulatory structure positively impacting AYA trial enrollment	‘Speed up our IRB!’
	Trial education	Communication and education of medical care providers and community about AYA focused trials and its importance	‘Increase knowledge in the community regarding our clinical trials’
4. How can the AYA COG RI Initiative foster successful accrual of AYA’s to clinical trials at your institution?	Clarify site enrollment procedures	Clear pathways at site level to allow enrollment of AYAs across cooperative groups	‘Make NCTN cross enrollment workflow clear to all MDs and CRAs’
	Enhance medical oncology engagement	Improved communication and relationship with medical oncology partners	‘Find strategies to reach out to adult oncologists about the benefits of enrollment on peds trials’
	Foster development of AYA trials	Advocating the need to open more AYA focused trials	‘Helping to voice the need for more available studies’
	Education of enrollment barriers	Exchange of information about barriers to enrollment at specific sites to all AYA RI members	‘Continued examples of strategies to address common obstacles’
	Disseminate information on trials	Increase knowledge about AYA trials within AYA RI network which leads to more AYAs enrolled on trials	‘A database could help with being able to identify trials that might be particularly useful for our population’
	Systemic process changes at NCTN/NCI	Initiate NCT/NCI level changes that allow for positive impact on AYA trial enrollment	‘Making the process of activating COG trials through NCTN as easy as possible’

The survey also asked participants to provide recommendations for how the COG AYA RI Network could further support local site’s efforts to increase AYA trial enrollment. A total of 75 responses from 60 respondents were reviewed and categorized. The level of agreement on categorization on first review was 84% and 93% at reconciliation. The remaining 7% were reconciled by a third reviewer to reach 100% consensus. Recommendations included providing education on enrollment barriers (33%), enhancing medical and pediatric oncology engagement (20%), disseminating information on trials (17%), fostering the development of AYA CCTs (15%), fostering process changes at the NCTN/NCI (10%) and clarifying site enrollment procedures (7%) (Fig. 2B). Specific examples are presented in Table 3.

## Discussion

In this survey of COG sites with designated AYA RIs, several shared barriers and facilitators to AYA enrollment were identified that appeared to be independent of institutional demographics and infrastructure. The most common shared barriers included poor communication between medical and pediatric oncology, hesitation of cross-enrollment onto cooperative group CCTs, administrative logistical barriers at the institutional level and perception of lack of available CCTs. Leading facilitators to enrollment reported by sites included strong communication between pediatric and medical oncology, supportive research infrastructure and presence of AYA champions.

Apart from logistical barriers, lower cross-enrollment by medical oncologists on COG trials was a major perceived institutional barrier. Limited knowledge of available COG CCTs, lack of time and resources and administrative hurdles (e.g., registering with COG, etc.) may be underlying reasons for poor cross-enrollment by medical oncologists, as documented in previous studies. Importantly, lack of available AYA CCTs is only partially responsible for lower cross-enrollment as some medical oncology programs with similar availability of AYA CCTs also had similar suboptimal accrual rates^14–16^. Although formal studies are lacking, reports from established AYA programs have hypothesized the benefits of establishing processes that enable medical oncologists and research teams to be aware of available trials and eligible patients^17,18^. Increasing the number of AYA champions and engaging medical oncologists in disease specific COG CCTs could be potential solutions. Also, where logistically feasible, research coordinators could be shared amongst pediatric and medical oncology provider groups such that familiarity with and knowledge of AYA CCTs is shared between groups.

Perceived lack of availability of AYA CCTs is a shared barrier and has been suggested by multiple prior studies^9^. Large national efforts, including the reorganization of NCTN which, by consolidating the network groups and supporting closer collaboration, fostered cross network enrollment, have been undertaken and are in process to address this barrier. In our survey, availability of AYA CCTs was often noted to be dependent on the primary oncology service, pediatric or medical oncology. The referral patterns, insurance contracts, administrative policies implementing existing age cut offs for obtaining cancer care largely determine where AYAs get their care and impact their treatment. AYA trials may be more likely to be opened by pediatric oncology sites compared with medical oncology sites^19,20^ and adolescents treated by adult medical oncologists are less likely to be enrolled in clinical trials^16^. The hospital treatment setting, community vs academic, also plays a major role. Over 90% of cancer patients under the age of 15 are treated at a tertiary care center versus less than 20% of 15–40-year-old cancer patients^21,22^. Community oncology practices, where the majority of AYAs are treated, often do not have access to AYA CCTs. Furthermore, there is often limited communication between the pediatric and medical oncologists in the community cancer care setting, and thus, there is often limited knowledge of locally available CCTs^21^. In addition, the 18-year-old lower age limit of eligibility continues to limit younger AYA participation in CCTs evaluating novel targeted therapies, including immunotherapy trials, and this needs to be further addressed at the national level^22^.

The current survey further identified potential areas for high-yield interventions to enhance enrollment. Based on this information, key targets for local intervention could include: (1) improving communication between pediatric and medical oncology; (2) employing AYA-specific personnel, such as a patient navigator; and (3) implementing an AYA CCT screening process. Robust communication between pediatric and medical oncology services was perceived as a strong facilitator to CCT enrollment. Increased interaction via tumor boards have been effective in fostering communication between different services and disciplines^23^. Tumor boards represent an opportunity to review open trials, identify eligible patients and identify optimal treatment approaches for patients with rare cancers and/or complicated presentations. Regular attendance and visibility of AYA team members at shared multidisciplinary tumor boards (MTBs) is crucial to establishing referral networks, enabling ongoing screening of eligible patients and facilitating enrollment^24^. One example is the EORTC-SPECTA, a virtually conducted molecular profiling MTB specifically focused on recruiting AYA patients with newly diagnosed relapsed high-grade gliomas and high-grade bone and soft tissue sarcomas. A virtual central pathology review with a clinically-validated molecular profiling report is provided to referring clinicians to improve access to novel drugs for AYAs^25^. Similarly, virtual MTBs may help knowledge sharing across disciplines and improve collaboration between providers and geographically-limited centers as evidenced during the ongoing pandemic^26^.

AYA programs also foster communication between pediatric and medical oncology. While mostly limited to single institution reports, AYA programs appear to be associated with improved CCT participation due to dedicated staff connecting AYA patients with CCTs^27–29^. However, the financial implications of developing an AYA program and allocating additional resources to clinical research are barriers. Philanthropic and institutional financial and non-financial support is often needed to launch such initiatives.

Patient navigators and supportive care professionals are well-poised to identify and address needs, values and communication styles of AYA cancer patients and survivors can serve as a conduit for identifying eligible patients for CCTs and relieve some responsibility from the primary medical team. One paramount role of the patient navigator is to serve as a first point of contact for the patient’s care team, with the ability to collaborate with other departments^30^. In this role, navigators can help bridge the knowledge gap of available AYA CCTs across pediatric and medical oncology departments, as is being studied by the AYA Program in Utah^31^.

The development of screening procedures to capture eligible AYAs can also enhance AYA CCT enrollment. Implementation of a standard operating procedure for screening at a cancer treatment site improved access and referral to NCTN AYA CCTs presenting to different oncology providers at an academic site. (verbal communication Grimes) Additional studies are needed to identify optimal screening procedures.

A framework for interventions based on the information obtained from the survey has been presented in Table 4. National opportunities to improve AYA enrollments were also highlighted. Maximizing the availability of AYA CCTs for local sites to open has the potential to increase enrollment in so far as the local site is interested in the study question and willing to open the trial. The development of cross-network AYA CCTs was an important step taken by NCTN to improve AYA accrual. While there have been some challenges to cross-enrolling AYAs on cross-network CCTs due to limited knowledge of the cross-enrollment process and differences in treatment approaches between pediatric and medical oncologists, many of these barriers are currently being addressed. The network groups across the NCTN are co-developing concepts at earlier stages of trial design and development and numerous efforts have been made to increase awareness of the cross-enrollment process, including the development of cross-network enrollment frequently asked questions. These efforts have led to a record number of AYA trials available through the NCTN and the number and diversity of these trials is rapidly increasing. Enrollment on these trials is also increasing, as evidenced by the SWOG-led Hodgkin lymphoma trial S1826, in which COG has enrolled more than 30% of the patients to date.

**Table 4:** Framework for barriers identified and changes desired and implemented.

Barriers expressed	Desired institutional change	Ways COG can foster this change	Interventions currently implemented or in process
Poor communication between medical oncology and pediatric oncology	Improve communication with medical oncology AYA Program with medical oncology AYA Navigator	Enhance Medical oncology engagement Educate regrading enrollment barriers Disseminate trial information	Include medical oncologists in AYA RI Network /Joint leadership NCTN cross-network trial development AYA RI network group webinars focusing on barriers and facilitators to enrollment
Administrative logistical barriers	Unified trial screening Expand age limit for pediatric oncology intake AYA Program Shared AYA Navigator	Foster development of AYA Trials Clarify site enrollment procedures	NCTN cross-network trial development
Cross-enrollment challenges	Unified trial screening Increased research resources	Clarify site enrollment procedures Systemic NCTN changes	Development of COG FAQ NCTN cross-network trial development
Complex COG trials	Increase education on trials	Educate and disseminate information on current trials	AYA RI Network webinars focused on individual AYA relevant trials
Trials not available	Increase research resources	Foster development of AYA Trials	NCTN cross-network trial development

While increasing the availability of studies is an important step to increasing enrollment, provider variability in practice and awareness may be more challenging to address at a national level given the diversity of clinical practices and patient populations. However, addressing provider hesitancy and lack of collaboration is critically important. Our survey suggests that education on AYA CCT enrollment processes and trial availability may help to overcome some of the provider hesitancy in offering CCTs to AYAs. The AYA RI Network and NCORP have developed efforts to directly address this gap in knowledge^10^. Through a series of webinars, these groups have sought to increase interaction amongst AYA oncology providers to disseminate information on available trials and provide guidance on overcoming local barriers to AYA CCT enrollment. Expanding pediatric and medical oncology provider participation in these webinars might be an opportunity to further increase the reach of these educational endeavors.

This study has a few limitations. The RI Network consists of individuals who have an interest in addressing disparities in AYA enrollment and are likely more aware about AYA trials and enrollment processes than their colleagues. Thus participant responses may not be fully representative of other stakeholders, particularly those at sites that are not participating in the AYA RI Network. Further, the RI Network consists mostly of pediatric oncologists, and while the current survey did not assess participants’ role, it is likely that the survey captured a limited number of perspectives from medical oncology stakeholders. It will be extremely important in future studies to obtain a broader perspective from medical oncologists working in varied practice settings on the barriers, enablers, and the feasibility of the proposed strategies to improve accrual. However, stakeholders representing the supportive disciplines and regulatory office were surveyed to capture different perspectives. In addition, the survey focused on medical oncologists cross-enrolling on COG trials and did not include perspectives on pediatric oncologists or medical oncologists enrolling AYA patients on other network group trials. Further studies evaluating medical oncology stakeholder perspectives are needed.

To our knowledge, the extent to which barriers and facilitators to AYA CCT enrollment are shared among institutions has not been previously reported. The shared barriers and facilitators identified in this study provide prime targets for potential intervention to improve enrollment. Studies addressing, and not just describing, the dismal enrollment of AYA CCT enrollment are urgently needed and our survey highlights starting points to begin to optimize the process.

## Supplementary Information

Supplementary Information 1.

Supplementary Information 2.
