# Supplementary material for: Shared barriers and facilitators to enrollment of adolescents and young adults on cancer clinical trials
Source: Sci Rep. 2022 Mar 9;12:3875. doi: 10.1038/s41598-022-07703-5 (PMC8907177; doi:10.1038/s41598-022-07703-5)
Supplement: Supplementary file 1 — Supplementary Information 1. [file 41598_2022_7703_MOESM1_ESM.docx]

Supplementary Table 1 – AYA RI Network survey tool

| 1. What are the main facilitators to accrual of AYA patients (ages 15-39) onto COG clinical trials at your institution? If applicable, please include facilitators to collaboration with medical oncology for clinical trial accrual in your response. 2. What are the main barriers to accrual of AYA patients onto COG clinical trials at your institution? If applicable, please include barriers to collaboration with medical oncology for clinical trial accrual in your response. 3. If you could change one thing at your institution to increase accrual of AYA patients to COG clinical trials, what would it be?      1. How can the AYA COG RI Initiative foster successful accrual of AYA’s to clinical trials at your institution?   **Demographics of program and institution**   1. Approximately how many AYAs are diagnosed at your institution each year?    1. ≤50    2. 51-100    3. 101-200    4. 201-500    5. 501-1000    6. >1000 2. Does your institution have an AYA Oncology program (a formal program that provides personalized services that address some or all of the unique medical and psychosocial needs of AYA cancer patients who are newly-diagnosed or are on active treatment)    1. Yes    2. No    3. In the process of development   If you answered ‘Yes’ or ‘In process of development’ to Question 1, please respond to questions 2-5 below. Otherwise, please move on to Question 6.   1. Which of the following services does your AYA Oncology program provide (select all that apply)    1. Cancer Treatment    2. Genetic Counseling    3. Oncofertility    4. Psychosocial support    5. Sexual Health    6. Survivorship care    7. Symptom management    8. Other _____________________________ 2. In the past 3 years, on average, how many AYA patients per year have been cared for by your AYA oncology program) 3. ≤50 4. 51-100 5. 101-150 6. 151-200 7. >200 8. Does your AYA program serve patients with specific cancer diagnoses (such as hematologic malignancies or sarcomas, etc) or is it open to all AYAs with any cancer diagnosis?    1. Our AYA program serves only AYAs with specific cancer diagnoses    2. Our AYA program cares for all AYAs with cancer, irrespective of their cancer diagnosis    3. Other – please describe 9. Which of the following descriptions best describes your pediatric site (select all that apply):    1. Free standing children’s hospital    2. Children’s hospital within larger medical center    3. Community-based hospital    4. Academic medical center/University affiliation    5. Other- please describe 10. Please describe the geographic proximity between medical oncology and pediatric oncology services at your institution     1. Pediatric and medical oncology work in the same building     2. Pediatric and medical oncology work on the same campus but are not located in the same building     3. Pediatric oncology and medical oncology work on separate campuses     4. Other, please describe 11. Does your division/department currently have regularly scheduled shared tumor boards with medical oncologists?     1. Yes, we have regularly scheduled tumor boards for **most** cancer diagnoses     2. Yes, we have regularly scheduled tumor boards for **some** cancer diagnoses     3. No 12. Does your division/department currently have ad hoc discussions with medical oncologists about specific AYA patient cases?     1. Yes, we have ad hoc discussions with medical oncology about AYA cases for **most** cancer diagnoses     2. Yes, we have ad hoc discussions with medical oncology about AYA cases for **some** cancer diagnoses     3. No 13. How often do medical oncologists at your institution/partnering adult hospital enroll their patients on COG clinical trials when eligible and available? 14. Very frequently 15. Frequently 16. Occasionally 17. Rarely 18. Never 19. We do not have medical oncologists at our institution or at a partnering hospital 20. Do you or any of your partners have privileges to see adults (age≥21) with cancer at your institution or at a partnering adult hospital/program? 21. Yes 22. No 23. Do medical oncologists at your institution/partnering adult hospital have COG membership and enroll their own patients onto COG trials? 24. Some medical oncologists are COG members (at least 1) and consent and enroll their patients onto COG trials 25. Some medical oncologists are COG members (at least 1), however, pediatric oncology consent and enroll their patients onto COG trials 26. Some medical oncologists are COG members (at least 1), however, their patients do not enroll onto COG trials 27. Medical oncologists are not COG members, however, pediatric oncology consent and enroll their patients on COG trials 28. Medical oncologists are not COG members, and pediatric oncologists do not enroll medical oncology patients onto COG trials 29. We do not have medical oncologists at our institution or at a partnering hospital 30. Other____________ 31. At your institution, is there a single IRB for approving both pediatric and medical oncology studies? 32. Yes 33. No 34. We do not have medical oncologists at our institution or at a partnering hospital |
| --- |
