# Supplementary material for: Shared barriers and facilitators to enrollment of adolescents and young adults on cancer clinical trials
Source: Sci Rep. 2022 Mar 9;12:3875. doi: 10.1038/s41598-022-07703-5 (PMC8907177; doi:10.1038/s41598-022-07703-5)
Supplement: Supplementary file 2 — Supplementary Information 2. [file 41598_2022_7703_MOESM2_ESM.pptx]

## Slide 1
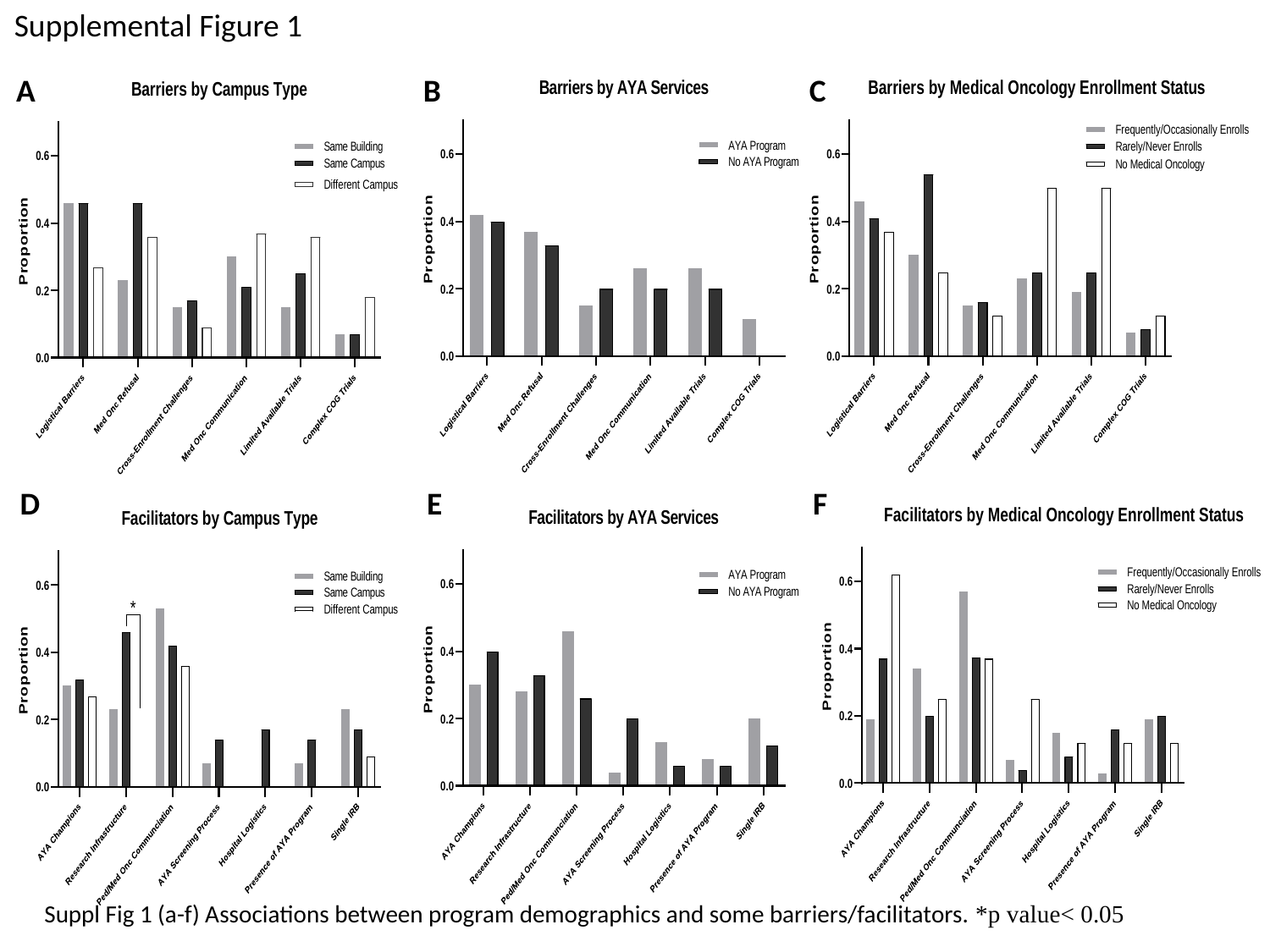

Supplemental Figure 1
C
A
B
F
D
E
Suppl Fig 1 (a-f) Associations between program demographics and some barriers/facilitators. *p value< 0.05
